# Can Linear Regression Modeling Help Clinicians in the Interpretation of Genotypic Resistance Data? An Application to Derive a Lopinavir-Score

**DOI:** 10.1371/journal.pone.0025665

**Published:** 2011-11-16

**Authors:** Alessandro Cozzi-Lepri, Mattia C. F. Prosperi, Jesper Kjær, David Dunn, Roger Paredes, Caroline A. Sabin, Jens D. Lundgren, Andrew N. Phillips, Deenan Pillay, for the EuroSIDA and the United Kingdom CHIC/United Kingdom HDRD Studies

**Affiliations:** 1 Department of Infection and Population Health, Division of Population Health, UCL Medical School, Royal Free Campus, London, United Kingdom; 2 Division of Biostatistics, University of Minnesota, Minneapolis, Minnesota, United States of America; 3 Department of Pathology, Immunology and Laboratory Medicine, Emerging Pathogens Institute, College of Medicine, University of Florida, Gainesville, Florida, United States of America; 4 Copenhagen HIV Programme, University of Copenhagen, Panum Institute, Copenhagen, Denmark; 5 Medical Research Council Clinical Trials Unit, London, United Kingdom; 6 HIV Unit and irsiCaixa Retrovirology Lab, Hospital Universitari Germans Trias i Pujol, Barcelona, Spain; 7 Centre for Viral Disease, Department of Infectious Diseases, Copenhagen University Hospital, Copenhagen, Denmark; 8 Department of Virology, University College London, London, United Kingdom; Institut National de la Santé et de la Recherche Médicale, France

## Abstract

**Background:**

The question of whether a score for a specific antiretroviral (e.g. lopinavir/r in this analysis) that improves prediction of viral load response given by existing expert-based interpretation systems (IS) could be derived from analyzing the correlation between genotypic data and virological response using statistical methods remains largely unanswered.

**Methods and Findings:**

We used the data of the patients from the UK Collaborative HIV Cohort (UK CHIC) Study for whom genotypic data were stored in the UK HIV Drug Resistance Database (UK HDRD) to construct a training/validation dataset of treatment change episodes (TCE). We used the average square error (ASE) on a 10-fold cross-validation and on a test dataset (the EuroSIDA TCE database) to compare the performance of a newly derived lopinavir/r score with that of the 3 most widely used expert-based interpretation rules (ANRS, HIVDB and Rega). Our analysis identified mutations V82A, I54V, K20I and I62V, which were associated with reduced viral response and mutations I15V and V91S which determined lopinavir/r hypersensitivity. All models performed equally well (ASE on test ranging between 1.1 and 1.3, p = 0.34).

**Conclusions:**

We fully explored the potential of linear regression to construct a simple predictive model for lopinavir/r-based TCE. Although, the performance of our proposed score was similar to that of already existing IS, previously unrecognized lopinavir/r-associated mutations were identified. The analysis illustrates an approach of validation of expert-based IS that could be used in the future for other antiretrovirals and in other settings outside HIV research.

## Introduction

The treatment of human immunodeficiency virus (HIV)-positive individuals with combination antiretroviral therapy (cART) has significantly reduced the morbidity and mortality associated with HIV [1], [2]. However, the development of antiretroviral-resistant HIV mutations remains one of the factors that can impair the efficacy of cART [3], [4]. Lopinavir/ritonavir (LPV/r), approved by the FDA in September 2000 and in Europe in April 2001, has been widely used in the management of treatment-experienced patients. It represents one of the options for initiation of treatment in antiretroviral-naïve patients in resource-rich countries and first choice for second-line treatment in resource-limited countries [5]–[7]. Prediction of the impact of specific patterns of protease mutations on the efficacy of ritonavir-boosted protease inhibitors (PI/r) is complicated, as clinically relevant resistance generally requires multiple mutations and can develop through the interplay of major and minor mutations in a variety of patterns. Several genotypic interpretation scores for LPV/r have been developed [8]–[14], but there is little consensus on their relative value and little attempt has been made to date to create a set of meta-rules standardized across interpretation systems (IS) [14]. In addition to these scores, clinicians often consult web-based IS such as the *Agence nationale de recherches sur le SIDA* (ANRS), REGA and Stanford IS. One of the analyses of the TITAN trial attempted to standardize the ‘cut-off levels’ used by seven currently proposed IS to divide patients into those likely to have or not to have reduced susceptibility to LPV/r and identified potentially more sensitive cut-offs, although in the specific context of deciding whether to use LPV/r or darunavir [15]. The common issue with these IS is that they are mainly expert-based and although very transparent about the mutations that are included, how each of them are weighted and why, typically weights are not derived using methods of statistical inference. In addition, the Stanford IS, for example, makes the implicit assumption that there is a marginal effect of each mutation for a specific drug which is the same, regardless of other concomitantly detected mutations (i.e. there are no interactions between mutations). These IS often differ in the mutations ascribed to reduce or enhance susceptibility. In general, the question of whether a score for specific antiretrovirals could be derived from analyzing the correlation between genotypic data and virological response using statistical methods which is superior to currently existing expert-based IS remains largely unanswered [16], [17]. The aim of this analysis was two-fold: i) to compare a small number of covariate selection strategies in the linear regression framework, known to perform well for prediction in the context of high dimensional data, ii) to use the best performing covariate selection method to derive a new LPV/r score and to compare its predictive value to that of available expert web-based IS. In order to achieve this objective, we used the data from two large, independent and well characterized cohort studies of HIV-positive individuals in Europe: the EuroSIDA cohort and the merged data of the UK Collaborative HIV Cohort (UK CHIC) Study and the UK HIV Drug Resistance Database (UK HDRD).

## Materials and Methods

### Dataset

Using the data of the patients in the EuroSIDA cohort and in the UK CHIC Study, for whom genotypic data were stored in the UK HDRD, we constructed a database of treatment change episodes (TCE) similar to those used in other previous collaborative studies [16]–[20]. Clinical data are collected in the 2 cohorts following rigid criteria which have been extensively described in detail elsewhere [21], [22]. Briefly, all viral loads as well as dates of starting and stopping all antiretroviral drugs are routinely collected in all patients enrolled in these cohorts. EuroSIDA requests that both genotypic tests performed at the clinical sites and plasma samples are collected prospectively. Please see Information S1 for study structure and contributing clinical sites in UK CHIC and EuroSIDA cohorts.

Retrospective genotypic sequencing has been carried out on samples identified for specific projects. HIV-1 RNA is isolated from patient blood plasma using QIAamp kit (Qiagen, Barcelona, Spain) and sequence analysis of HIV-1 RT and PR reading frames is performed using the Trugene HIV-1 genotyping Kit and OpenGene DNA Sequencing System according to the manufacturer's recommendations (Bayer, Barcelona, Spain). Data on resistance for the patients included in the UK CHIC cohort are obtained through the linkage with the UK HDRD, which contains information on genotypic resistance tests performed on behalf of most HIV clinics in the UK. Mutations are identified by comparison against a reference sequence of the subtype B isolate, HXB2 in both databases.

All patients provided written consent to participate to UK CHIC and EuroSIDA, following procedures in accordance with the ethical standards of the responsible committee on human experimentation and the Helsinki Declaration. Ethics approval (for the use of the databases) from the Institutional Review Board (IRB) at all institutions/hospitals where participants were recruited and human experimentation was conducted was obtained. No specific consent for inclusion in the current analysis was needed.

**Figure 1:**
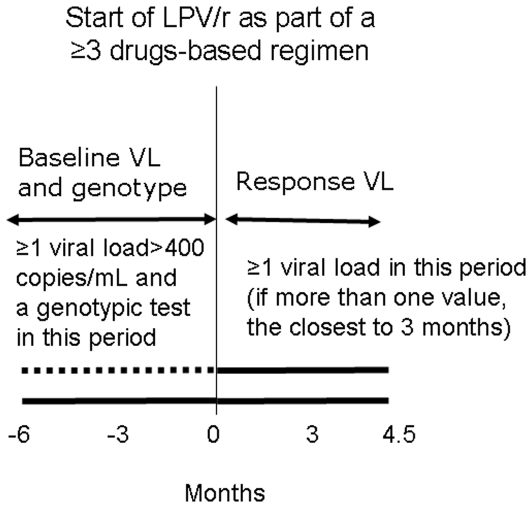
Description of a lopinavir-based TCE.

A TCE entailed any change in therapy in which a patient initiated LPV/r with a viral load >400 copies/mL as part of a combination including ≥2 antiretrovirals (cART) (although we refer to “treatment change episodes” it should be noted that initiations of lopinavir/r-based cART from ART-naïve patients are also included in this analysis and TCE could entail simply the addition of LPV/r to a failing regimen). For each TCE we recorded: all drugs initiated together with LPV/r, all drugs currently received as part of cART, the viral load and the results of a genotypic resistance test performed in the 6 months preceding the initiation of LPV/r and a follow-up viral load measured over the first 4 months from starting LPV/r. When multiple baseline or follow up data were available, the value closest to month 3 after initiation of LPV/r was used (Figure 1). Because only a minority of patients (<5%) contributed more than one TCE (more than one distinct combination of these 3 key TCE-defining features existed at 2 or 3 time points) no attempt in the analysis was made to correct for violation of independence of individual observations. TCE including drugs not belonging to the 3 original major drug classes (NRTI, NNRTI and PI) were not considered because only RT and PR regions of HIV were sequenced. Using the results of the genotypic tests we could generate a genotypic susceptibility score (GSS) for all antiretrovirals started together with LPV/r using the rules of ANRS IS (version 19) [23]. We also derived the lopinavir predictions using the 3 most common expert-opinion base IS (ANRS v19, Rega v8.0.2 [24] and Stanford v6.0.10 [25]). To make predictions of Stanford comparable to the other 2 web-based IS we grouped “potential low-level resistance” with “susceptible” and “low-level resistance” with “intermediate”. For this analysis, the UK CHIC/UK HDRD databases provided the training/validation datasets (n = 1,174 TCEs) and EuroSIDA provided the test dataset (n = 388 TCEs).

### Statistical analysis

The main characteristics of the TCE were described after stratification by cohort study. Because it is generally impossible to know, a priori, which off-the-shelf machine learning/statistical approaches will perform best for a given prediction problem and data set, we used a standard linear regression model with interaction terms for simplicity. The outcome was the change in viral load from pre-TCE to post-TCE levels on the log_10_ scale. For patients whose viral load decreased to undetectable levels we used the naïve approach of replacing the unobserved undetectable value with the limit of detection of the assay used. In sensitivity analyses, we instead replaced the unobserved undetectable value with ½ of the limit or with the fixed value of 20 copies/mL.

The basic set of covariates (pre-LPV/r start viral load, exact month of viral load response (ranging between 1 and 4 months) and the ANRS predictions for the other drugs started besides LPV/r were considered in all regression models but were not forced into a model unless they were found to improve the prediction performance according to the specific selection criterion used. We then constructed 4 separate models including: i) the LPV/r Rega GSS, ii) the LPV/r ANRS GSS, and iii) the LPV/r Stanford GSS. All web-based GSS were fitted as categorical variables with “susceptible” as the reference group. Model iv) included individual PI mutations and 2-way interactions between these rather than a specific susceptibility score. Candidate PI mutations to be included were those reported as being a major mutation with non-zero prevalence associated with PI-resistance in either the IAS-USA December 2010 [26] list or any of the considered web-based IS and minor mutations which were detected with a prevalence >5% (see complete list in the Results).

Three different criteria for the selection of mutations and 2-way interactions between mutation terms were used -best subset least squared estimations (LSE), least absolute shrinkage and selection operator (LASSO) [27] and a hybrid version of least angle regression (LAR) and LASSO. The hybrid method is a modification of the LAR originally proposed by Efron et al. [28]. In this approach, the sequence of models is determined by the original LAR algorithm but the coefficients of the parameters for the model at any step are determined using ordinary least squares. Both LASSO and LAR are shrinkage and selection methods for linear regression which minimize the usual sum of squared errors though with a bound on the sum of the absolute values of the coefficients given by a complexity parameter (Table S2). This parameter was chosen to minimize the average squared error (ASE) based on a tenfold cross-validation (CV) on the UK CHIC/UK HDRD TCE database. Briefly, 10-fold CV works by dividing the dataset randomly into ten equal parts. The method fits the model for a range of values of the complexity parameter to nine-tenths of the data and then computes the prediction error on the remaining one-tenth. This is done, in turn, for each one-tenth of the data, and eventually the 10 prediction error estimates are averaged. From this procedure we obtained an estimated prediction of the 10-fold CV error (CV PRESS) curve as a function of the model evolution steps which was used to establish where to stop the inclusion of the covariates. In practical terms, the “one-standard-error” rule was used by picking the most parsimonious model within one standard error of the minimum CV PRESS. In contrast, the training set is used to determine the coefficients but not to decide when to stop as the CV PRESS in training decreases monotonically at each step regardless of the number of steps.

Cross-validation was applied to the UK CHIC/UK HDRD database (training/validation set) to select the complexity parameter for both LASSO and the hybrid version of LAR and LASSO. In contrast, the EuroSIDA database (test set) was never used for training but to judge the performance of the selected models. We identified the mutations marginally associated with the outcome first and then fitted a separate model incorporating all 2-way interactions among this subset of mutations only. Although the categorical variables for the predictions of ANRS, Rega and Stanford IS were forced to remain in the corresponding models, the other parameters to be included (from pre-TCE viral load, GSS of other drugs and exact months from TCE to post-TCE viral load) were selected using CV.

The performance of the models was tested by comparing the magnitude of the ASE on the test dataset using analysis of variance (ANOVA) with robust empirical estimates of the standard errors. For completeness the R-Square on training and the ASE on both training and validation were also shown. In addition, we transformed the continuous outcome into a binary variable (using the cut-off of reduction of >1.5 log copies/mL), calculated the accuracy (i.e. the percentage of patients correctly classified) and performed a likelihood ratio test to compare these percentages by model from fitting a GEE Poisson regression model. All analyses were performed using the procedure (for CV), MIXED (for ANOVA) and GENMOD (for GEE) in SAS 9.2 (SAS Institute, Cary, NC, USA, 2010).

## Results

Table 1 shows the main characteristics of the TCE stratified by training and test set. These characteristics were extremely similar in the two cohorts. Viral load decreased by an average of 2.0 log_10_ copies/mL upon initiation of LPV/r. There was a small percentage (<3%) of truncated changes in viral load due to the response viral load being measured with an assay with lower limit of 400 copies/mL and a substantially higher censoring below 50 copies/mL (30%). The majority of patients did not just add LPV/r to an existing regimen but started a complete new regimen with an average of 2 new nucleosides as well as LPV/r. The NRTI most frequently used was lamivudine (45%). Fewer than 10% of patients also started a NNRTI (mainly efavirenz). Median calendar year of TCE was 2003 (range:1998–2008). Only 21 (5%) of the EuroSIDA patients and 35 (3%) of those from UK CHIC contributed 2 TCEs.

**Table 1:** Description of viral load and treatment in the TCE database stratified by cohort.

Characteristics	Dataset	
	UK CHIC/UK HDRD	EuroSIDA
	N = 1174	N = 388
**Pre-TCE viral load, log10 copies/mL**		
Median (range)	4.63 (2.61, 7.18)	4.52 (2.63, 6.33)
**Post-TCE viral load, log10 copies/mL**		
Median (range)	2.00 (1.70, 6.15)	2.22 (0.78, 6.31)
**Viral load reduction, log10 copies/mL**		
Median (range)	2.17 (−2.43, 5.21)	1.93 (−1.80, 4.60)
**% censored below 400 copies/mL, n(%)**	26 (2.2%)	11 (2.8%)
**% censored below 50 copies/mL, n(%)**	440 (37.5%)	92 (23.7%)
**Time from TCE to post-TCE viral load, months**		
Median (range)	3 (1, 4)	3 (1, 4)
**NRTI in regimen at time of TCE**		
zidovudine	312 (26.6%)	78 (20.1%)
stavudine	214 (18.2%)	79 (20.4%)
lamivudine	565 (48.1%)	173 (44.6%)
emtrcitabine	85 (7.2%)	12 (3.1%)
tenofovir	576 (49.1%)	136 (35.1%)
didanosine	391 (33.3%)	154 (39.7%)
abacavir	340 (29.0%)	150 (38.7%)
**NNRTI in regimen at time of TCE**		
efavirenz	125 (10.6%)	73 (18.8%)
nevirapine	78 (6.6%)	32 (8.2%)
etravirine	5 (0.4%)	3 (0.8%)
**PI in regimen at time of TCE**		
saquinavir-HG	59 (5.0%)	23 (5.9%)
saquinavir-SG	10 (0.9%)	19 (4.9%)
indinavir	11 (0.9%)	30 (7.7%)
ritonavir	1174 (100%)	388 (100%)
amprenavir	26 (2.2%)	33 (8.5%)
atazanavir	14 (1.2%)	5 (1.3%)
darunavir	0 (0.0%)	1 (0.3%)
nelfinavir	15 (1.3%)	3 (0.8%)
**No. new drugs started at time of TCE**		
Median (range)	4 (3, 8)	3 (3, 7)
**NRTI newly started at time of TCE**		
zidovudine	276 (23.5%)	74 (19.1%)
stavudine	146 (12.4%)	67 (17.3%)
lamivudine	466 (39.7%)	119 (30.7%)
emtrcitabine	82 (7.0%)	12 (3.1%)
tenofovir	511 (43.5%)	129 (33.2%)
didanosine	299 (25.5%)	140 (36.1%)
abacavir	285 (24.3%)	107 (27.6%)
**NNRTI newly started at time of TCE**		
efavirenz	109 (9.3%)	75 (19.3%)
nevirapine	61 (5.2%)	31 (8.0%)
etravirine	5 (0.4%)	3 (0.8%)
**PI newly started at time of TCE**		
saquinavir-HG	39 (3.3%)	24 (6.2%)
saquinavir-SG	10 (0.9%)	19 (4.9%)
indinavir	6 (0.5%)	28 (7.2%)
ritonavir	1174 (100%)	388 (100%)
amprenavir	26 (2.2%)	33 (8.5%)
atazanavir	14 (1.2%)	4 (1.0%)
darunavir	0 (0.0%)	1 (0.3%)
nelfinavir	3 (0.3%)	3 (0.8%)

Table 2 shows the extent of PI-resistance captured by the genotype at the time of the TCE. A minority of patients show mutations associated with major resistance to LPV/r. The most prevalent of these mutations was V82F, detected in 5–8% of patients respectively in UK CHIC/HDRD and EuroSIDA. This lower prevalence was also reflected in the IS predictions indicating, on average, that approximately 90% of patients carried a virus which was susceptible to LPV/r regardless of the system used. Over 50% of TCE include 2 other newly initiated drugs which were predicted to be active by the ANRS IS. Among the more polymorphic PI mutations (nevertheless included in the IAS-USA December 2010 list as PI-resistance mutations) the most prevalent ones were E35D, M36I, L63P and V91S reaching a percentage ranging between 30% and 50%.

**Table 2:** Description of HIV drug resistance prior to TCE stratified by cohort.

HIV resistance	Dataset	
	UK CHIC/UK HDRD	EuroSIDA
	N = 1174	N = 388
**Mutations in HIV protease, n(%)**		
L10I	194 (16.5%)	61 (15.7%)
I13V	346 (29.5%)	52 (13.4%)
I15V	306 (26.1%)	49 (12.6%)
G16E	87 (7.4%)	3 (0.8%)
K20I	91 (7.8%)	15 (3.9%)
K20R	84 (7.2%)	38 (9.8%)
**V32I**	6 (0.5%)	10 (2.6%)
E35D	437 (37.2%)	65 (16.8%)
M36I	476 (40.5%)	80 (20.6%)
M46I	46 (3.9%)	43 (11.1%)
**I47A**	0 (0.0%)	0 (0.0%)
**I47V**	4 (0.3%)	7 (1.8%)
I54V	56 (4.8%)	34 (8.8%)
D60E	86 (7.3%)	16 (4.1%)
I62V	283 (24.1%)	80 (20.6%)
L63P	593 (50.5%)	120 (30.9%)
I64V	212 (18.1%)	36 (9.3%)
H69K	294 (25.0%)	18 (4.6%)
A71V	116 (9.9%)	53 (13.7%)
A71T	77 (6.6%)	17 (4.4%)
**L76V**	1 (0.1%)	2 (0.5%)
V77I	322 (27.4%)	49 (12.6%)
**V82A**	4 (0.3%)	8 (2.1%)
**V82F**	63 (5.4%)	32 (8.2%)
**V82T**	4 (0.3%)	10 (2.6%)
**V82S**	1 (0.1%)	1 (0.3%)
I84V	38 (3.2%)	23 (5.9%)
L89M	251 (21.4%)	16 (4.1%)
L90M	117 (10.0%)	64 (16.5%)
V91S	428 (36.5%)	89 (22.9%)
**IS predictions, n(%)**		
**ANRS lop/r**		
Susceptible	1129 (96.2%)	339 (87.4%)
Intermediate	36 (3.1%)	30 (7.7%)
Resistant	9 (0.8%)	19 (4.9%)
**Rega lop/r**		
Susceptible	1115 (95.0%)	333 (85.8%)
Intermediate	49 (4.2%)	43 (11.1%)
Resistant	10 (0.9%)	12 (3.1%)
**Stanford lop/r**		
Susceptible	1045 (89.0%)	306 (78.9%)
Intermediate	114 (9.7%)	72 (18.6%)
Resistant	15 (1.3%)	10 (2.6%)
**ANRS GSS other drugs (predicted no. of active)**		
0	32 (2.7%)	30 (7.7%)
0.5	46 (3.9%)	13 (3.4%)
1	253 (21.6%)	44 (11.3%)
1.5	63 (5.4%)	16 (4.1%)
2	651 (55.5%)	218 (56.2%)
2.5	14 (1.2%)	1 (0.3%)
3	102 (8.7%)	55 (14.2%)
>3	13 (1.1%)	11 (2.8%)

In bold major IAS-USA December 2010 mutations for lopinavir/r.

**Figure 2:**
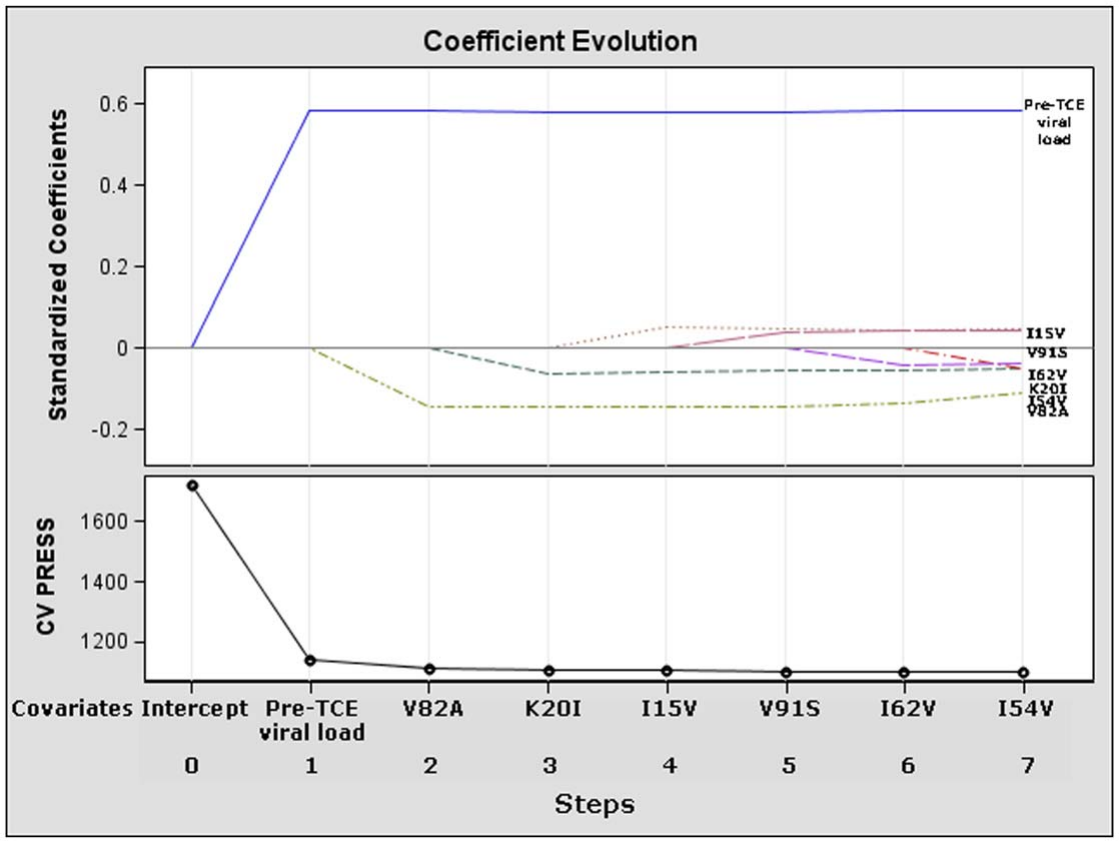
Plot of the standardized coefficients of all the factors selected at each step (from step 1 to final step 7) of the best subset (LSE) method are plotted as a function of the step number. This enables to assess the relative importance of each factor selected at any step of the selection process as well as provides information as to when effects entered the model. The lower plot in the panel shows how CV PRESS (the criterion used to choose the selected model) changes as factors enter or leave the model. Selection was halted at step 7 when the “one-standard error” rule was achieved.

When we used the best subset selector, LSE estimates of the coefficients and 10-fold CV to identify our model potentially leading to a novel LPV/r score, besides pre-TCE viral load, 5 mutations were selected: I15V (parameter estimate = +0.13), K20I (estimate = −0.26), I54V (estimate = −0.29), I62V (estimate = −0.11) V82A (estimate = −0.60) and V91S (estimate = +0.11). Figure 2 shows the relative importance of these factors at each step of the selection process as well as provides information as to when effects entered the model (the estimates -or weights- above are derived directly from the standardized coefficients at final step 7 and are summarized in Table 3, a negative weight means a negative impact on viral response).

**Table 3:** Coefficients (standard errors) associated with covariates included in the model.

Interpretation system	Mutations, susceptibility scores and interaction effects coefficient (se)											
	I15V	K20I	I54V	I62V	V82A	V91S	I62V*V82A	I15V*V82A	VL*I54V	VL*V82A	I	R
**Model i)** ANRS											−.74 (0.18)	−1.46 (0.37)
**Model ii)** Rega											−.61 (0.16)	−1.74 (0.34)
**Model iii)** Stanford											−.29 (0.11)	−1.38 (0.27)
**Model iv)** Best subset LSE main effects	+.13 (0.07)	−.26 (0.13)	−.29 (0.18)	−.11 (0.08)	−.60 (0.17)	+.11 (0.07)						
Best subset LSE main effects+2 ways interactions	+.10 (0.07)	−.24 (0.12)	−.28 (0.18)	−.10 (0.08)	+1.89 (0.77)	+.13 (0.07)	−.51 (0.34)	−.13 (0.07)				
LASSO main effects			−.12 (0.08)		−.36 (0.21)							
LASSO viral load+2 ways interactions									−.07 (0.01)	−.13 (0.08)		
LAR/LASSO main effect					−.80 (0.15)							
LAR/LASSO viral load+2 ways interaction										−.17 (0.07)		

I = Intermediate, R = Resistant.

In contrast, the mutations included in the interpretation rules for LPV/r of the 3 expert-based IS are shown in Table S1. Figure S1 shows the evolution of the ASE at each step until final step 7 was reached and inclusion of factors terminated because the ten error estimates computed by cross-validation (CV PRESS) stopped showing a decrease using the “one-standard-error” rule. Step 7 was the chosen closing step for most of other fit criteria considered (including AIC, BIC or adjusted R-square, Figure S2).

The ASE was greatly reduced when pre-TCE viral load entered the model and additionally reduced when V82A entered the model. The improvements in ASE after the introduction of viral load is a key finding as it implies that the models including viral load are a substantial improvement compared to the null model (with intercept only) but that adding some of the genotypic information further improves the predictive performance. Only minor changes of the ASE were seen after further introducing the other mutations on the training, validation, and test datasets. Note that while, as expected, the ASE on the training data decreased monotonically, the ASE on the validation set (the portions of UK CHIC/HDRD which were not used for training) started to slightly increase beyond step number four. This indicates that the models after step 4 were beginning to overfit the training data. The ASE on the test set was a lot larger than the validation ASE although there was no sign of over-fitting.

Interestingly, neither the GSS for other drugs started with LPV/r nor the exact month of viral load response were retained in the final model of main effects. Two 2-way interactions were identified to improve the fit of the models: I62V/A82V (weight of I62V decreased to −0.51 when A82V was not detected) and I15V/A82V (weight of I15V increased to +0.50 when A82V was not detected, likelihood ratio test p = 0.0001). However, the introduction of these terms did not lead to a further decrease in ASE (Table 4).

**Table 4:** Average squared error, R-squares and accuracy according to selection criteria on the training, validation, and test datasets at final inclusion step.

	Training		Validation	Test (EuroSIDA)	
Interpretation system	ASE	R-Square	ASE	ASE*	Accuracy**
**Model i)** ANRS	1.037	0.354	1.207	1.299	0.655
**Model ii)** Rega	1.074	0.359	0.786	1.258	0.647
**Model iii)** Stanford	1.059	0.336	1.048	1.295	0.655
**Model iv)** Best subset LSE main effects	1.032	0.370	1.124	1.330	0.657
Best subset LSE main effects+2 ways interactions	1.018	0.379	1.132	1.338	0.662
LASSO main effects	1.063	0.352	1.109	1.315	0.650
LASSO main effects+2 ways interactions	1.041	0.365	1.130	1.326	0.650
LAR/LASSO main effects	1.051	0.359	1.135	1.374	0.655
LAR/LASSO main effects+2 ways interactions	1.044	0.363	1.136	1.372	0.655

*ANOVA p-value for the difference between models p = 0.34.

**Percentage correctly classified as successes (viral load drop >1.5 log copies/mL) or failures (viral load drop ≤1.5 log copies/mL); likelihood ratio test p-value from fitting a GEE model p = 0.98.

Both the LASSO and the LAR/LASSO models only include a maximum of 3 main effects: pre-TCE viral load and mutation V82A (LAR) and pre-TCE viral load and mutations I54V and V82A (LASSO) as well as their interactions with viral load. Table 4 shows the comparison between the final ASE across the models considered. All models seemed to perform equally well with little difference in the ASE although the Rega algorithm seemed to perform better both in validation and test datasets in the specific cross-validation shown (ANOVA p-value on test p = 0.34). A difference in ASE of 0.037 between Rega and Stanford corresponds to a difference in root mean square error (RMSE) of 0.19, meaning that the error in predictions (calculated as predicted minus observed) between the two systems was of 0.19 log copies/mL. Of note, the ASE on the test set was always larger than the ASE on the validation set regardless of the model used. For completeness, Table 4 also reports ASE and R-Squares in training. As expected on average, the derived scores tended to perform better in training although this tendency was reversed in the validation and test set comparisons. All models seem to be able to correctly classify 65% of participants and all performed equally (p = 0.98). A sensitivity analysis conducted after replacing unobserved undetectable viral load values with ½ of the limit of lower detection or with the fixed value of 20 copies/mL, provided similar results (data not shown).

## Discussion

Increasingly complex prediction methods are used to try to improve prediction of viral load response, in the light of existing expert-based IS. The aim of this analysis was to assess a likely incremental benefit of one of these more sophisticated approaches. The availability of a well characterized TCE database extracted from 2 large cohorts of HIV-positive patients in Europe gave us the opportunity to explore some relatively novel methodologies for the construction of statistically-based IS such as LAR and LASSO in the context of linear regression.

The first main result of our analysis is that in our example of lopinavir-based TCE the performance of a model based on linear regression was similar to that obtainable by currently available largely expert-opinion based IS. If anything, the use of the Rega IS seemed to provide better predictions on both validation and test datasets than linear regression even when predictors were selected with the more efficient LASSO criterion. Similar conclusions were previously drawn by other authors performing almost identical analyses although not in the context of LPV/r resistance and using a binary viral load outcome [17], [18]. In practical terms, after transforming the continuous outcome into a binary variable indicating failure (≤1.5 log copies/mL viral reduction from baseline) or success (>1.5 reduction) all models seemed to be able to correctly classify 65% of the participants.

A second main result was the fact that adding interaction terms to our model did not improve the predictions. This finding has potentially two implications: the assumption made by web-expert opinions (e.g. Stanford) that there is no interaction between mutations for LPV/r and that mutations can be given a single weight for their marginal effect may be valid. Indeed, in our analysis whilst we found evidence that the weight for mutation I62V should be different according to whether A82V was also detected, the incorporation of this information did not lead to an improvement in predictions. Although not proved by this analysis, the second implication is that other commonly used non-parametric non-linear approaches (such as random forests or neural networks) are also unlikely to lead to improved performance in this specific analysis; indeed, preliminary work based on this dataset (data not shown) and others had already suggested this point [17]. Of note, the exact time of the post-cART viral load measurement was a covariate which was not retained in any of the final models. A possible interpretation of this finding is that the viral drop upon initiation of a lopinavir-containing regimen is similar regardless of whether it is measured 1 month or up to 4 months from starting the drug, consistent with the view that it is a potent protease inhibitor.

Third, the results derived from cross-validation only tell part of the story. This is because, as previously shown, TCEs coming from a single source tend to be more similar to each other than TCEs coming from completely distinct settings [19]. In our analysis this was clearly shown by the large increase of the ASE in the test dataset (consisting of patients from EuroSIDA) as opposed to validation dataset (patients from the UK CHIC Study) and suggests an inherent limitation of statistical-derived IS aiming at predicting response in individuals who did not provide data for training. Reasons for this discrepancy are unclear. In the analysis by Assoumou et al [19], some of the variability seemed to be explained by differences in the treatment history of the populations enrolled in the different cohorts, which influenced the baseline viral genotypes and by antiretroviral treatment strategies which differed from one country to another. However, we did not detect large differences between UK CHIC and EuroSIDA in these parameters. It can also be argued that both EuroSIDA and UK CHIC are themselves an amalgam of data from diverse clinical sites although collected in a similar standardized way. The external validation using the EuroSIDA database (a completely independent dataset) is an important feature of this analysis which has rarely been employed in previous similar analyses [17], [29].

Several LPV/r scores have been constructed in recent years. One of the first LPV/r mutation scores was developed by comparing the genotypes and phenotypes of 112 viral isolates from early clinical trials [8]. Mutations at 11 amino acid positions in protease were found to be correlated with increased phenotypic resistance to LPV/r. Another weighted score was developed using 1,482 clinical samples available from the Monogram Biosciences database, for which both a genotype and phenotype for LPV/r had been performed [9] and validated in another analysis [10]. This score consists of 29 mutations at 18 protease positions and viruses with a score ≥7 were considered to have reduced susceptibility to LPV/r. Although this score was based on many more isolates than the LPV/r mutation score, the investigators were limited to making genotype-phenotype correlations. A large database from France (the ATU database) was used to evaluate the virological response to LPV/r in 792 treatment-experienced individuals and generated a LPV/r score [11]. Within the ATU dataset, mutations at 10 amino acid positions in protease were found to better predict virological response than the previously described scores. Some authors proposed and showed the potential utility of genotypic inhibitory quotients (GIQ) based on the ratio between LPV/r concentration and number of LPV/r resistance mutations) [30]–[32] although due to lack of consensus on which list of mutations to use, the predictive value of GIQ varies according to the IS used [32].

Regarding the mutations included in our LPV/r score, besides mutations I15V and V91S which seemed to be not currently recognized polymorphisms associated with a better response to LPV/r, V82A is listed by IAS-USA December 2010 as a major LPV/r mutation and used in the rules of all previously developed scores (Abbott 2007 score [12] ANRS and Rega IS [23], [24] and Food and Drug Association (FDA) LPV/r mutations list [13]). In the Stanford IS this mutation is currently given a weight of +25 which is the 2^nd^ highest weight, after that given to I47A (weight = +50) while the Rega IS assign a weight of 0.6, remarkably similar to ours but about half the weight given to I54V (Table S1). Interestingly, mutation V82A was the first to be entered in our model leading to the largest decrease in ASE besides pre-TCE viral load while V82F which was more prevalent than V82A was not selected at all. Mutations K20I and I54V were given smaller weights in our score which is consistent with the fact that are not major IAS-USA mutations but are listed in most available IS (I54V is not included in ANRS). Finally I62V seemed to have a marginal negative impact on viral response, especially when detected in the absence of V82A. Unfortunately we do not have phenotypic evidence to support this finding (it is scored as zero in all web-based IS –Table S1- and whilst sometimes associated with resistance to saquinavir or fosamprenavir [23] seems to be quite polymorphic). Of note, the weight of I62V was shrunk to zero by the LASSO procedure suggesting that this was a spurious association due to chance alone.

Some limitations of this analysis need to be discussed. First of all, in a setting in which viral load measures are truncated by the lower limit of the assay, binary outcomes are typically preferred. Although the percentage of censored response viral load was relatively low we may have introduced bias by replacing this unobserved value with the lower limit of detection of the assay. However, when we performed a sensitivity analysis by replacing the unobserved undetectable viral load values with ½ of the limit of lower detection or with the fixed value of 20 copies/mL results were similar. We did not choose to evaluate a wider range of statistical approaches for three main reasons: i) the performance of specific approaches is likely to vary according to the specific features of the dataset analyzed and therefore a unique “best predictive model” may not exist; ii) previous attempts to identify the best approach have reached discordant conclusions [17], [18], [20] and iii) linear regression is simple to implement in SAS (a robustly validated statistical package), easy to interpret and has been previously shown to have high predictive power in the field of HIV resistance [33]. The prevalence of some of the mutations considered key for lopinavir/r by expert opinion (e.g. V32I, I47V, L76V) was relatively low and this could have influenced the covariate selection for our score. Furthermore, we have only considered PI mutations previously found to be associated with reduced susceptibility to PI, thus we cannot exclude the possibility that our model could be outperformed by another one constructed using all changes from consensus in the PR region. Nevertheless, this addition would add further complexity and it is beyond the scope of the current exercise. We aim to repeat this analysis using a larger dataset including all major cohort studies in Europe. Similarly, one of the limitations of predictive systems is that population sequence assays only detect mutations present in major quasi-species. Indeed it has been shown that the inclusion of other information coming from ultra-deep sequencing, phenotypic data, genetic barrier of resistance, phylogenetic-type analyses or even drug history can significantly improve the predictive capability of a given model [28], [34]–[39]. Unfortunately besides drug history, which was not considered, none of these data are yet available in our cohorts. Other unmeasured factors such as adherence, known to be associated with the level of resistance, could have confounded the association between specific mutations and outcome.

LPV/r was for quite some time the standard boosted PI for treatment-experienced patients with PI-related resistance mutations. However, with the approval of other drugs such as tipranavir and particularly darunavir and atazanavir/r, it has become increasingly important for clinicians to be able to choose between these newer PI/r and LPV/r for these patients. Our analysis does not directly address the question of which PI/r should be used and in which circumstances, but suggests that the choice of LPV/r can successfully rely on currently available web-based IS predictions.

In conclusion, we fully explored the potential of linear regression to construct a simple predictive model for LPV/r-based TCE. Two of the identified mutations (V82A ad I54V) in our score are recognized LPV/r mutations listed by widely used expert-based IS. In addition, we identified previously unrecognized mutations I62V ad K20I, which appear to have a modest impact in reducing viral response, and mutations I15V and V91S which, in contrast, seem to determine LPV/r hypersensitivity, all of which need further investigation. Although, the performance of our proposed score seems to be similar to that of already existing IS, the same approach of validation of web-based IS could be used in the future for other antiretrovirals ideally including a larger number of TCE and in other settings outside HIV research.

## Supporting Information

Figure S1 **Evolution of the average squared error (ASE) on the training, validation, and test datasets when using the best subset selector and LSE estimators for the coefficients over the 7 steps at which a new factor has been introduced in the model.**
(TIF)

Figure S2 **Sequence of models and stopping step according to model fit statistics used; the sum of the 10 predicted residual sum of squares from the cross-validation (CV PRESS) was the criterion chosen for this analysis.**
(TIF)

Table S1 **Mutations included in the interpretation rules for lopinavir/r according to the 2010 IAS-USA list 3 widely used expert-based IS.** We highlighted those selected for our score.
(DOC)

Table S2 **A simple explanation of the least absolute shrinkage and selection operator (LASSO).**
(DOC)

Information S1 **Study structure and contributing clinical sites in UK CHIC and EuroSIDA cohorts.**
(DOC)
