# Supplementary figures and images for: Can Linear Regression Modeling Help Clinicians in the Interpretation of Genotypic Resistance Data? An Application to Derive a Lopinavir-Score
Source: PLoS One. 2011 Nov 16;6(11):e25665. doi: 10.1371/journal.pone.0025665 (PMC3217925; doi:10.1371/journal.pone.0025665)

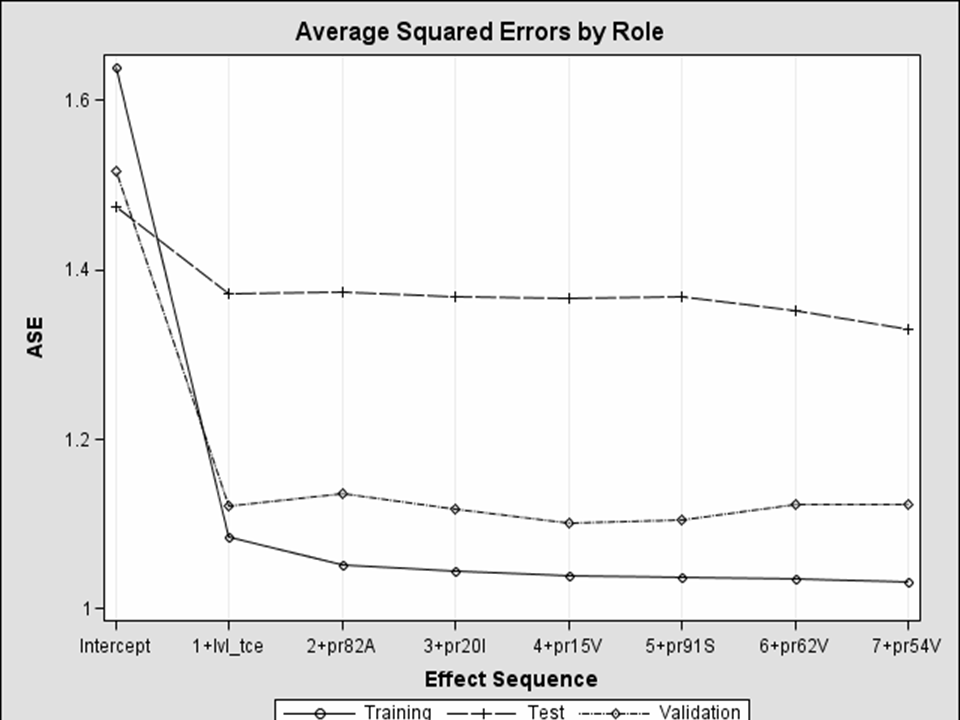

Supplement: Figure S1 — Evolution of the average squared error (ASE) on the training, validation, and test datasets when using the best subset selector and LSE estimators for the coefficients over the 7 steps at which a new factor has been introduced in the model. (TIF) [file pone.0025665.s001.tif]

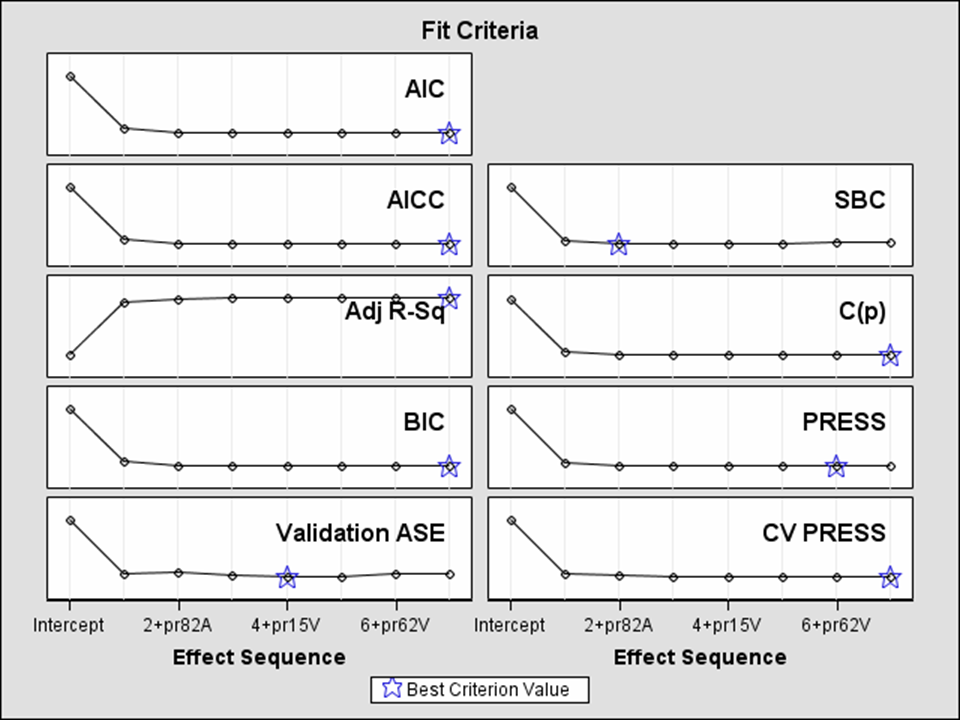

Supplement: Figure S2 — Sequence of models and stopping step according to model fit statistics used; the sum of the 10 predicted residual sum of squares from the cross-validation (CV PRESS) was the criterion chosen for this analysis. (TIF) [file pone.0025665.s002.tif]
