# Supplementary material for: Can Linear Regression Modeling Help Clinicians in the Interpretation of Genotypic Resistance Data? An Application to Derive a Lopinavir-Score
Source: PLoS One. 2011 Nov 16;6(11):e25665. doi: 10.1371/journal.pone.0025665 (PMC3217925; doi:10.1371/journal.pone.0025665)
Supplement: Table S1 — Mutations included in the interpretation rules for lopinavir/r according to the 2010 IAS-USA list 3 widely used expert-based IS. We highlighted those selected for our score. (DOC) [file pone.0025665.s003.doc]

**Table S1.** Mutations included in the interpretation rules for lopinavir/r according to the 2010 IAS-USA list 3 widely used expert-based IS. We highlighted those selected for our score.

| **Interpretation System** | Lopinavir/r mutations  (major in bold) our weight |
| --- | --- |
| **IAS-USA list 2010** | L10FIRV  K20MR  L24I  **V32I**  L33F  M46IL  **I47AV**  I50V  F53L  I54VLAMTS 0.29  L63P  A71VT  G73S  **L76V**  **V82AFTS** 0.60  I84V  L90M |
| **ANRS V19** | L10F/I/R/V  K20M/R  L24I  L33F  M46I/L  I50V  F53L  I54M/L/T/V 0.29  L63P  A71I/L/V/T  V82A/F/S/T 0.60  I84V  L90M  I47A  L76V |
| **Rega v8.0.2**  **(in brackets weights)** | I47A (+2)  I50V (+1.5)  I54AT (+1.5)  L76V (+1.5)  I47V (+1)  G48M (+1)  I54SV (+1) 0.29  V82FS (+1)  I84A (+1)    L10F (+0.5)  K20T (+0.5)  L24FI (+0.5)  V32I (+0.5)  L33F (+0.5)  K43T (+0.5)  M46IL (+0.5)  G48AV (+0.5)  F53L (+0.5)  I54LM (+0.5)  A71I (+0.5)  G73STC (+0.5)  V82ALMT (+0.5) 0.60  I84V (+0.5)  N88D (+0.5)  L90M (+0.5)    L10IV (+0.25)  K20IMRV (+0.25) 0.26  L33I (+0.25)  K43R (+0.25)  I64MV (+0.25)  A71TV (+0.25)  V77ATI (+0.25)  I50L (-0.25) |
| **Stanford HIVD v 6.0.10**  **(in brackets the weights)** | L10F (+4)  L10I (+2)  L10R (+2)  L10V (+2)  L10Y (+2)  L24I (+3)  V32I (+12)  L33F (+5)  K43T (+2)  M46I (+10)  M46L (+10)  M46V (+5)  I47V (+15)  I47A (+50)  G48V (+10)  G48M (+10)  G48A (+5)  G48S (+5)  G48T (+5)  G48Q (+5)  G48E (+5)  I50V (+20)  I50L (-5)  F53L (+3)  F53Y (+2)  I54L (+12)  I54M (+12)  I54S (+12)  I54T (+12)  I54V (+12) 0.29  I54A (+12)  A71T (+2)  A71V (+2)  A71I (+2)  A71L (+2)  G73C (+2)  G73S (+2)  G73T (+2)  G73A (+2)  T74P (+5)  L76V (+25)  V82A (+25)  V82F (+25)  V82S (+25)  V82T (+25)  V82M (+10)  V82L (+10)  V82C (+10)  I84A (+15)  I84V (+15)  I84C (+15)  L89V (+2)  L90M (+10) |
