# Supplementary material for: Can Linear Regression Modeling Help Clinicians in the Interpretation of Genotypic Resistance Data? An Application to Derive a Lopinavir-Score
Source: PLoS One. 2011 Nov 16;6(11):e25665. doi: 10.1371/journal.pone.0025665 (PMC3217925; doi:10.1371/journal.pone.0025665)
Supplement: Table S2 — A simple explanation of the least absolute shrinkage and selection operator (LASSO). (DOC) [file pone.0025665.s004.doc]

**Table S2.**

**A simple explanation of the least absolute shrinkage and selection operator (LASSO)**

Give a set of input measurements x1, x2 ...xp and an outcome measurement y,

the LASSO fits a linear model

yhat=b0 + b1*x1+ b2*x2 + ... +bp*xp

The criterion used is:

Minimize sum( (y-yhat)^2 ) subject to sum[absolute value(bj)] ≤λ j=0,1,2,….,p

The first sum is taken over all observations in the dataset. The bound "λ" is the complexity parameter. When "λ" is large enough, the constraint has no effect and the solution is just the usual multiple linear least squares regression of y on x1, x2, ...xp.

However when for smaller values of λ (λ≥0) the solutions are shrunken versions of the least squares estimates (LSE). Often, some of the coefficients bj are zero. Choosing "λ" is like choosing the number of predictors to use in a regression model, and cross-validation is a good tool for estimating the best value for "λ".
