# Supplementary material for: Can Linear Regression Modeling Help Clinicians in the Interpretation of Genotypic Resistance Data? An Application to Derive a Lopinavir-Score
Source: PLoS One. 2011 Nov 16;6(11):e25665. doi: 10.1371/journal.pone.0025665 (PMC3217925; doi:10.1371/journal.pone.0025665)
Supplement: Information S1 — Study structure and contributing clinical sites in UK CHIC and EuroSIDA cohorts. (DOC) [file pone.0025665.s005.doc]

# UK CHIC

***Steering Committee:*** Jonathan Ainsworth, Jane Anderson, Abdel Babiker, David Dunn, Philippa Easterbrook, Martin Fisher, Brian Gazzard (Chair), Richard Gilson, Mark Gompels, Teresa Hill, Margaret Johnson, Clifford Leen, Chloe Orkin, Andrew Phillips, Deenan Pillay, Kholoud Porter, Caroline Sabin, Tariq Sadiq, Achim Schwenk, Nicky Mackie, Alan Winston, Valerie Delpech.

***Central Co-ordination****: Medical Research Council Clinical Trials Unit (MRC CTU), London* (David Dunn, Adam Glabay, Kholoud Porter); *Royal Free NHS Trust and RFUCMS, London* (Loveleen Bansi, Teresa Hill, Andrew Phillips, Caroline Sabin).

***Participating Centres:*** *Barts and The London NHS Trust, London* (Chloe Orkin, Kevin Jones, Rachel Thomas); *Brighton and Sussex University Hospitals NHS Trust* (Martin Fisher, Nicky Perry, Anthony Pullin, Duncan Churchill); *Chelsea and Westminster NHS Trust, London* (Brian Gazzard, Steve Bulbeck, Sundhiya Mandalia, Jemima Clarke); *Health Protection Agency – Centre for Infections London (HPA)* (Valerie Delpech); *Homerton University Hospital NHS Trust, London* (Jane Anderson, Sajid Munshi); *King’s College Hospital, London* (Philippa Easterbrook, Frank Post, Yasar Khan, Paragi Patel, Fatimah Karim, Stephen Duffell); *Medical Research Council Clinical Trials Unit (MRC CTU), London* (Abdel Babiker, David Dunn, Adam Glabay, Kholoud Porter); *Mortimer Market Centre, Royal Free and University College Medical School (RFUCMS), London* (Richard Gilson, Shuk-Li Man, Ian Williams); *North Middlesex University Hospital NHS Trust, London* (Achim Schwenk); *Royal Free NHS Trust and RFUCMS, London* (Margaret Johnson, Mike Youle, Fiona Lampe, Colette Smith, Helen Grabowska, Clinton Chaloner, Dewi Ismajani Puradiredja, Loveleen Bansi, Teresa Hill, Andrew Phillips, Caroline Sabin); *St. Mary’s Hospital, London* (Nicky Mackie, Alan Winston, Jonathan Weber, Christian Kemble, Mark Carder); *The Lothian University Hospitals NHS Trust, Edinburgh* (Clifford Leen, Alan Wilson); *North Bristol NHS Trust* (Mark Gompels, Debbie Dooley).

**UK HIV Drug Resistance Database**

**Steering Committee:**

Jane Anderson, Homerton University Hospital, London; David Asboe and Anton Pozniak, Chelsea & Westminster Hospital, London; Sheila Burns, Royal Infirmary of Edinburgh; Sheila Cameron, Gartnavel General Hospital, Glasgow; Patricia Cane, Health Protection Agency, Porton Down; Ian Chrystie, Guy’s and St. Thomas’ NHS Foundation Trust, London; Duncan Churchill, Brighton and Sussex University Hospitals NHS Trust; Duncan Clark, St Bartholomew's and The London NHS Trust; Valerie Delpech and Deenan Pillay, Health Protection Agency, Centre for Infections, London; Linda Lazarus, Expert Advisory Group on AIDS Secretariat, Health Protection Agency, London; David Dunn, Esther Fearnhill, Hannah Green and Kholoud Porter, MRC Clinical Trials Unit, London; Philippa Easterbrook and Mark Zuckerman, King’s College Hospital, London; Anna Maria Geretti, Royal Free NHS Trust, London; Paul Kellam, Deenan Pillay, Andrew Phillips and Caroline Sabin, Royal Free and University College Medical School, London; David Goldberg, Health Protection Scotland, Glasgow; Mark Gompels, Southmead Hospital, Bristol; Antony Hale, Leeds Teaching Hospitals NHS Trust; Steve Kaye, St. Mary’s Hospital, London; Svilen Konov, Community Advisory Board; Andrew Leigh-Brown, University of Edinburgh; Nicola Mackie, St. Mary’s Hospital, London; Chloe Orkin, St. Bartholomew's Hospital, London; Erasmus Smit, Health Protection Agency, Birmingham Heartlands Hospital; Peter Tilston, Manchester Royal Infirmary; Ian Williams, Mortimer Market Centre, London; Hongyi Zhang, Addenbrooke’s Hospital, Cambridge

**Participating laboratories:**

Addenbooke’s Hospital, Cambridge (Hongyi Zhang); Department of Virology, St Bartholomew’s and The London NHS Trust (Duncan Clark, Ines Ushiro-Lumb, Tony Oliver, David Bibby); Belfast Health and Social Care Trust (Suzanne Mitchell); HPA Birmingham Public Health Laboratory (Erasmus Smit); Chelsea and Westminster Hospital, London (Adrian Wildfire); Dulwich Hospital, London (Melvyn Smith); Royal Infirmary of Edinburgh (Jill Shepherd); West of Scotland Specialist Virology Lab Gartnavel, Glasgow (Alasdair MacLean); Guy’s and St. Thomas’ NHS Foundation Trust, London (Ian Chrystie); Leeds Teaching Hospitals NHS Trust (Diane Bennett); Specialist Virology Centre, Liverpool (Mark Hopkins) and Manchester (Peter Tilston); Department of Virology at Royal Free Hospital, London (Clare Booth, Ana Garcia-Diaz); St Mary’s Hospital, London (Steve Kaye); University College London Hospitals (Stuart Kirk).

The UK HIV Drug Resistance Database is partly funded by the Department of Health; the views expressed in the publication are those of the authors and not necessarily those of the Department of Health. Additional support is provided by Bristol-Myers Squibb, Gilead, Pfizer, and Tibotec (a division of Janssen-Cilag Ltd).

This project was supported by NEAT (European Commission NEAT contract FP6/03757; NEAT is the acronym of European AIDS Treatment Network)

Primary support for Alessandro Cozzi-Lepri for this work (statistical analysis) was provided by the European Commission NEAT program.

**The EuroSIDA Study Group**The multi-centre study group o­n EuroSIDA (national coordinators in parenthesis).

**Argentina:** (M Losso), C Elias, Hospital JM Ramos Mejia, Buenos Aires.

**Austria:** (N Vetter), Pulmologisches Zentrum der Stadt Wien, Vienna; R Zangerle, Medical University Innsbruck, Innsbruck.

**Belarus:** (I Karpov), A Vassilenko, Belarus State Medical University, Minsk, VM Mitsura, Gomel State Medical University, Gomel; O Suetnov, Regional AIDS Centre, Svetlogorsk.

**Belgium:** (N Clumeck), S De Wit, M Delforge, Saint-Pierre Hospital, Brussels; R Colebunders, Institute of Tropical Medicine, Antwerp; L Vandekerckhove, University Ziekenhuis Gent, Gent.

**Bosnia-Herzegovina:** (V Hadziosmanovic), Klinicki Centar Univerziteta Sarajevo, Sarajevo.

**Bulgaria:** (K Kostov), Infectious Diseases Hospital, Sofia.

**Croatia:** (J Begovac), University Hospital of Infectious Diseases, Zagreb.

**Czech Republic:** (L Machala), H Rozsypal, Faculty Hospital Bulovka, Prague; D Sedlacek, Charles University Hospital, Plzen.

**Denmark:** (J Nielsen), G Kronborg,T Benfield, M Larsen, Hvidovre Hospital, Copenhagen; J Gerstoft, T Katzenstein, A-B E Hansen, P Skinhøj, Rigshospitalet, Copenhagen; C Pedersen, O D Larsen, Odense University Hospital, Odense; L Ostergaard, Skejby Hospital, Aarhus.

**Estonia:** (K Zilmer), West-Tallinn Central Hospital, Tallinn; Jelena Smidt, Nakkusosakond Siseklinik, Kohtla-Järve.

**Finland:** (M Ristola), Helsinki University Central Hospital, Helsinki.

**France:** (C Katlama), Hôpital de la Pitié-Salpétière, Paris; J-P Viard, Hôpital Necker-Enfants Malades, Paris; P-M Girard, Hospital Saint-Antoine, Paris; JM Livrozet, Hôpital Edouard Herriot, Lyon; P Vanhems, University Claude Bernard, Lyon; C Pradier, Hôpital de l'Archet, Nice; F Dabis, D Neau, Unité INSERM, Bordeaux.

**Germany:** (J Rockstroh), Universitäts Klinik Bonn; R Schmidt, Medizinische Hochschule Hannover; J van Lunzen, O Degen, University Medical Center Hamburg-Eppendorf, Infectious Diseases Unit, Hamburg; HJ Stellbrink, IPM Study Center, Hamburg; S Staszewski, JW Goethe University Hospital, Frankfurt; J Bogner, Medizinische Poliklinik, Munich; G. Fätkenheuer, Universität Köln, Cologne.

**Greece:** (J Kosmidis), P Gargalianos, G Xylomenos, J Perdios, Athens General Hospital; G Panos, A Filandras, E Karabatsaki, 1st IKA Hospital; H Sambatakou, Ippokration Genereal Hospital, Athens.

**Hungary:** (D Banhegyi), Szent Lásló Hospital, Budapest.

**Ireland:** (F Mulcahy), St. James's Hospital, Dublin.

**Israel:** (I Yust), D Turner, M Burke, Ichilov Hospital, Tel Aviv; S Pollack, G Hassoun, Rambam Medical Center, Haifa; S Maayan, Hadassah University Hospital, Jerusalem.

**Italy:** (S Vella), Istituto Superiore di Sanità, Rome; R Esposito, I Mazeu, C Mussini, Università Modena, Modena; C Arici, Ospedale Riuniti, Bergamo; R Pristera, Ospedale Generale Regionale, Bolzano; F Mazzotta, A Gabbuti, Ospedale S Maria Annunziata, Firenze; V Vullo, M Lichtner, University di Roma la Sapienza, Rome; A Chirianni, E Montesarchio, M Gargiulo, Presidio Ospedaliero AD Cotugno, Monaldi Hospital, Napoli; G Antonucci, F Iacomi, P Narciso, C Vlassi, M Zaccarelli, Istituto Nazionale Malattie Infettive Lazzaro Spallanzani, Rome; A Lazzarin, R Finazzi, Ospedale San Raffaele, Milan; M Galli, A Ridolfo, Osp. L. Sacco, Milan; A d’Arminio Monforte, Istituto Di Clinica Malattie Infettive e Tropicale, Milan.

**Latvia:** (B Rozentale), I Zeltina, Infectology Centre of Latvia, Riga.

**Lithuania:** (S Chaplinskas), Lithuanian AIDS Centre, Vilnius.

**Luxembourg:** (R Hemmer), T Staub, Centre Hospitalier, Luxembourg.

**Netherlands:** (P Reiss), Academisch Medisch Centrum bij de Universiteit van Amsterdam, Amsterdam.

**Norway:** (V Ormaasen), A Maeland, J Bruun, Ullevål Hospital, Oslo.

**Poland:** (B Knysz) J Gasiorowski, Medical University, Wroclaw; A Horban, E Bakowska, Centrum Diagnostyki i Terapii AIDS, Warsaw; A Grzeszczuk, R Flisiak, Medical University, Bialystok; A Boron-Kaczmarska, M Pynka, M Parczewski, Medical Univesity, Szczecin; M Beniowski, E Mularska, Osrodek Diagnostyki i Terapii AIDS, Chorzow; H Trocha, Medical University, Gdansk; E Jablonowska, E Malolepsza, K Wojcik, Wojewodzki Szpital Specjalistyczny, Lodz.

**Portugal:** (F Antunes), M Doroana, L Caldeira, Hospital Santa Maria, Lisbon; K Mansinho, Hospital de Egas Moniz, Lisbon; F Maltez, Hospital Curry Cabral, Lisbon.

**Romania:** (D Duiculescu), Spitalul de Boli Infectioase si Tropicale: Dr. Victor Babes, Bucarest.

**Russia:** (A Rakhmanova), Medical Academy Botkin Hospital, St Petersburg; E Vinogradova, St Petersburg AIDS Centre, St Peterburg; S Buzunova, Novgorod Centre for AIDS, Novgorod.

**Serbia:** (D Jevtovic), The Institute for Infectious and Tropical Diseases, Belgrade.

**Slovakia:** (M Mokráš), D Staneková, Dérer Hospital, Bratislava.

**Slovenia:** (J Tomazic), University Clinical Centre Ljubljana, Ljubljana.

**Spain:** (J González-Lahoz), V Soriano, P Labarga, J Medrano, Hospital Carlos III, Madrid; S Moreno, Hospital Ramon y Cajal, Madrid; B Clotet, A Jou, R Paredes, C Tural, J Puig, I Bravo, Hospital Germans Trias i Pujol, Badalona; JM Gatell, JM Miró, Hospital Clinic i Provincial, Barcelona; P Domingo, M Gutierrez, G Mateo, MA Sambeat, Hospital Sant Pau, Barcelona.

**Sweden:** (A Karlsson), Venhaelsan-Sodersjukhuset, Stockholm; L Flamholc, Malmö University Hospital, Malmö.

**Switzerland:** (B Ledergerber), R Weber, University Hospital, Zürich; P Francioli, M Cavassini, Centre Hospitalier Universitaire Vaudois, Lausanne; B Hirschel, E Boffi, Hospital Cantonal Universitaire de Geneve, Geneve; H Furrer, Inselspital Bern, Bern; M Battegay, L Elzi, University Hospital Basel.

**Ukraine:** (E Kravchenko), N Chentsova, Kiev Centre for AIDS, Kiev; V Frolov, G Kutsyna, Luhansk State Medical University; Luhansk; S Servitskiy, Odessa Region AIDS Center, Odessa; M Krasnov, Kharkov State Medical University, Kharkov.

**United Kingdom:** (S Barton), St. Stephen's Clinic, Chelsea and Westminster Hospital, London; AM Johnson, D Mercey, Royal Free and University College London Medical School, London (University College Campus); A Phillips, MA Johnson, A Mocroft, Royal Free and University College Medical School, London (Royal Free Campus); M Murphy, Medical College of Saint Bartholomew's Hospital, London; J Weber, G Scullard, Imperial College School of Medicine at St. Mary's, London; M Fisher, Royal Sussex County Hospital, Brighton; C Leen, Western General Hospital, Edinburgh.
 **Virology group:** B Clotet, R Paredes(Central Coordinators) plus ad hoc virologists from participating sites in the EuroSIDA Study.

**Steering Committee:**  F Antunes, B Clotet, D Duiculescu, J Gatell, B Gazzard, A Horban, A Karlsson, C Katlama, B Ledergerber (Chair), A D’Arminio Montforte, A Phillips, A Rakhmanova, P Reiss (Vice-Chair), J Rockstroh

**Coordinating Centre Staff:** J Lundgren, O Kirk, A Mocroft, A Cozzi-Lepri, D Grint, M Ellefson, D Podlekareva, J Kjær, L Peters, J Reekie, J Kowalska, J Tverland, A H Fischer, J Nielsen
